# Establishment of Stable Knockdown of MACC1 Oncogene in Patient-Derived Ovarian Cancer Organoids

**DOI:** 10.3390/mps7060104

**Published:** 2024-12-20

**Authors:** Sophia Hierlmayer, Liliia Hladchenko, Juliane Reichenbach, Christoph Klein, Sven Mahner, Fabian Trillsch, Mirjana Kessler, Anca Chelariu-Raicu

**Affiliations:** 1 Department of Obstetrics and Gynecology, University Hospital, Ludwig-Maximilians-University Munich, 81377 Munich, Germany; sophia.hierlmayer@campus.lmu.de (S.H.); l.hladchenko@campus.lmu.de (L.H.); juliane.reichenbach@med.uni-muenchen.de (J.R.); sven.mahner@med.uni-muenchen.de (S.M.); fabian.trillsch@med.uni-muenchen.de (F.T.); anca.chelariu-raicu@med.uni-muenchen.de (A.C.-R.); 2 Bavarian Cancer Research Center (BZKF), 81377 Munich, Germany; 3 Department of Pediatrics, Dr. Von Hauner Children’s Hospital, University Hospital, Ludwig Maximilians University Munich, 80337 Munich, Germany; christoph.klein@med.uni-muenchen.de; 4 German Cancer Consortium, DKTK, Partner Site Munich, 69120 Heidelberg, Germany

**Keywords:** ovarian cancer patient-derived organoids, lentiviral transduction, MACC1, oncogenic mechanisms, knockdown validation

## Abstract

High-grade serous ovarian cancer (HGSOC) remains the most lethal gynecological malignancy, and there is still an unmet medical need to deepen basic research on its origins and mechanisms of progression. Patient-derived organoids of high-grade serous ovarian cancer (HGSOC-PDO) are a powerful model to study the complexity of ovarian cancer as they maintain, in vitro, the mutational profile and cellular architecture of the cancer tissue. Genetic modifications by lentiviral transduction allow novel insights into signaling pathways and the potential identification of biomarkers regarding the evolution of drug resistance. Here, we provide an in-depth and detailed protocol to successfully modify the gene expression of HGSOC-PDOs by lentiviral transduction. As an example, we validate our protocol and create a stable knockdown of the MACC1 oncogene with an efficacy of ≥72% in two HGSOC-PDO lines, which remained stable for >3 months in culture. Moreover, we explain step-by-step the sample preparation for the validation procedures on transcriptional (qPCR) and protein (Western Blot) levels. Sustained downregulation of specific genes by lentiviral transduction enables the analysis of the resulting phenotypic and morphological changes. It serves as a valuable in-vitro model to study the mechanisms of ovarian cancer pathogenesis and allows for the evaluation of therapeutic approaches.

## 1. Introduction

Epithelial ovarian cancer (EOC) continues to rank among the most lethal gynecological malignancies, essentially due to its late detection in the advanced stages and relative resistance to conventional treatment strategies [1]. The development of patient-derived organoid (PDO) models has made it possible to study the complexity of EOC and offer a more accurate representation of tumor biology as they closely resemble the phenotypic profile of the cancer ex vivo [2,3].

In this article, we present a protocol for lentiviral transduction of EOC organoids, with a particular focus on the knockdown of the metastasis-associated in colon cancer 1 (MACC1) gene in representative high-grade serous ovarian cancer (HGSOC) lines, the most common and most aggressive form of EOC. MACC1 plays an important role in tumor progression and metastasis in a variety of human cancer types, including ovarian, pancreatic, lung, colorectal, and cholangiocarcinoma cancers [4,5,6,7,8]. It was shown in previous studies that the knockdown of MACC1 in pancreatic cancer cells decreases the liver metastatic lesions in a liver metastasis model [9]. In epithelial ovarian cancer cells, it has been shown that MACC1 inhibition increases cisplatin sensitivity in cells that had previously demonstrated cisplatin resistance [10]. Still, more research is needed to delineate and characterize the function of MACC1 in the context of ovarian cancer tissue.

The knockdown of MACC1 in HGSOC organoids aims to test its relevance in disease progression and its potential as a therapeutic target. The purpose of this study is to advance the functional genomic tools available for HGSOC research by improving the lentiviral transduction process. With the example of generating stable shRNA MACC1 organoid lines, this approach can be expanded to any customized shRNA sequence.

## 2. Related Work

Successful genetic manipulation of patient-derived organoids hedges the stable growth potential of the lines in the long-term culture. We have previously developed culture conditions and biobanking strategies to achieve robust long-term growth (>6 months) of ovarian cancer PDOs [11,12,13,14]. Lentiviral transduction of organoids, initially established in gastrointestinal mouse models [15], has also been previously reported for diverse human cancer and healthy tissue models; among others, these include fallopian tube organoids as well as ovarian cancer organoid lines (reporter construct) [3,12]. Still, experimental conditions to achieve a stable and sustainable shRNA knockdown of the gene of interest in high-grade serous ovarian cancer (HGSOC-PDO) have not been described in detail until now.

Here, we provide a comprehensive protocol for organoid preparation, transduction, and validation to obtain a shRNA-mediated stable knockdown maintained for more than 3 months in culture.

## 3. Protocol Design

This protocol uses HGSOC organoid lines, generated from solid tumor deposits of ovarian cancer patients, as host tissues for the introduction of a shRNA plasmid cassette. To facilitate transduction efficiency, this protocol foresees singularization of the organoids to a single-cell suspension, followed by incubation of the lentiviral particles and primary cells on a BME Type 2 coated surface. This approach allows for more natural virus-to-cell contact, avoiding centrifugation, thereby reducing the cellular stress before transfer to 3D. For full instructions and guidance on basic organoid cultivation and biobanking, please refer to Trillsch et al. [11].

**Figure 1:**
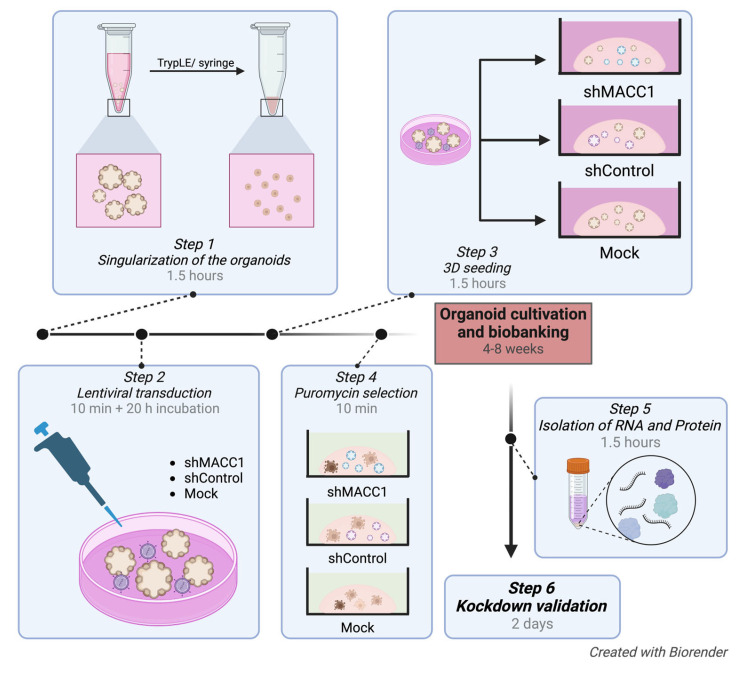
Overview of the experimental stages of lentiviral transduction of ovarian cancer organoids.

We describe, in detail, this experimental procedure (Figure 1, Table 1, Table 2 and Table 3) and demonstrate its efficiency in the example of knocked-down (KD) MACC1 in two organoid lines. Moreover, we describe downstream validation of RNA and protein levels to verify and quantify the KD efficacy.

As previously described for other cancer organoids [16], the transduction procedure poses some general methodological challenges, which we were aware of and would like to address. First, viral infection of organoids remains an invasive process and inevitably causes stress on the organoids. The mitigating steps described in this protocol do help preserve the viability of the culture, and observed cell death was comparable to other gene-modifying methods (e.g., nucleofection). However, if the gene of interest is involved in the regulation of stemness, this may lead to a reduction in growth or even failure to re-form organoids after transduction. In this case, inducible constructs might be a more appropriate strategy to consider. Third, depending on the potency of the shRNA as well as the place of integration, the efficacy of the knockdown might be variable. Nevertheless, the protocol is suitable for the routine establishment of knockdown organoid lines for either several genes in the isogenic background (with different selection cassettes) or a single gene in numerous lines within a relatively short timeframe (~1 month), which creates an opportunity to design and perform mechanistic studies in ovarian cancer organoid models in a well-controlled fashion.

## 4. Materials

### 4.1. Materials for Lentiviral Transduction

**Table 1:** Overview of the material needed for lentiviral transduction.

Substance	Company	Catalog Number
Advanced DMEM/F-12 Medium (ADF)	Gibco, Thermo Scientific, Waltham, MA, USA	12634028
GlutaMAX	Thermo Scientific, Waltham, MA, USA	35050038
HEPES (1 M)	Gibco, Thermo Scientific, Waltham, MA, USA	156630080
BME Type 2 cultivation matrix f.e. Cultrex Reduced Growth Factor Basement Membrane Extract, Type 2, Pathclear	R&D Systems, Minneapolis, MI, USA	3533-005-02
TrypLE Express Enzyme	Gibco, Thermo Scientific, Waltham, MA, USA	12604-013
Phosphate buffered saline	Gibco, Thermo Scientific, Waltham, MA, USA	10010023
Polybrene	Sigma-Aldrich^®^, Merck, Darmstadt, Germany	TR-1003-G
Puromycin	Gibco, Thermo Scientific, Waltham, MA, USA	A1113803
MISSION^®^ Lentiviral Transduction Particles, SHCLNV	Sigma-Aldrich^®^, Merck, Darmstadt, Germany	Ref. Seq.: NM_182762, TRC Clone ID: TRCN0000242547
MISSION^®^ pLKO.1-puro Non-Mammalian shRNA Control Transduction Particles	Sigma-Aldrich^®^, Merck, Darmstadt, Germany	SHC002V
TrypLE	Gibco, Thermo Scientific, Waltham, MA, USA	12604021

### 4.2. Materials for Knockdown Validation

**Table 2:** Overview of the material needed for knockdown validation.

Substance	Company	Catalog Number
β-Mercaptoethanol	Sigma-Aldrich^®^, Merck, Darmstadt, Germany	102343186
RLT-Buffer	QIAGEN, Hilden, Germany	79216
2 × Laemmli Buffer	Bio-Rad Laboratories GmbH, Feldkirchen, Germany	1610737
TaqMan^®^ Gene Expression Assays, MACC1	Applied Biosystems™, Thermo Scientific™, Waltham, MA, USA	4331182, Hs00766186_m1
TaqMan^®^ Gene Expression Assays, GAPDH	Applied Biosystems™, Thermo Scientific™, Waltham, MA, USA	4331182, Hs99999905_m1
TaqMan^®^ Universal PCR Master Mix	Applied Biosystems™, Thermo Scientific™, Waltham, MA, USA	1258699
Anti-MACC1 Antibody	Sigma-Aldrich^®^, Merck, Darmstadt, Germany	HPA020081
Anti-β-Actin Antibody	Sigma-Aldrich^®^, Merck, Darmstadt, Germany	A5441
Anti-Rabbit-HRP Antibody	Invitrogen, Thermo Scientific™, Waltham, MA, USA	G-21234
Anti-Mouse-HRP Antibody	Invitrogen, Thermo Scientific™, Waltham, MA, USA	G-2104
RNeasy Mini Kit	QIAGEN, Hilden, Germany	74104
cDNA Synthesis Kit	Biozym Scientific GmbH, Hessisch Oldendorf, Germany	331475
Nuclease Free water	Thermo Scientific, Waltham, MA, USA	A1113803
Bovine serum albumin	Sigma-Aldrich^®^, Merck, Darmstadt, Germany	A9647
Powdered Milk	Carl Roth GbmH & Co. KG, Karlsruhe, Germany	T145.2
0.2 µM pore-sized PVDF	Bio-Rad Rad Laboratories GmbH, Feldkirchen, Germany	1704156
Amersham ECL Prime Western Blotting Detection Reagent	Cytiva, Marlborough, MA, USA	RPN2232

### 4.3. Equipment

**Table 3:** Overview of tthe equipment needed.

Equipment	Company	Catalog Number
Biosafety level 2 facility	-	-
Centrifuge	Any brand	-
37 °C cell culture incubator	Any brand	-
Microscope	Any brand	-
pluriStrainer mini 200 µM	pluriSelect Life Science, Leipzig, Germany	43-10200-40
Falcon 24-well Polystyrene	Corning, Berlin, Germany	351447
Countesse 3 (optional Neubauer chamber)	Invitrogen, Thermo Scientific™, Waltham, MA, USA	AMQAX2000
NanoPhotometer Pearl	Implen, Munich, Germany	-
Applied Biosystems™ 7500 Fast Real-Time PCR System	Applied Biosystems™, Thermo Scientific™, Waltham, MA, USA	1704150
Trans-Blot^®^ Turbo™ Transfer System	Bio-Rad Rad Laboratories GmbH, Feldkirchen, Germany	1704150
Low Protein bind Eppendorf Tube	Eppendorf SE, Hamburg, Germany	0030108116
ChemiDoc MP Imaging System	Bio-Rad Laboratories GmbH, Feldkirchen, Germany	12003154

### 4.4. High-Grade Serous Ovarian Cancer Organoid Lines

- HGSOC_6 and HGSOC_8: organoid lines from LMU organoid biobank generated from chemo-naive, primary, advanced ovarian cancer tumor tissue at the time of debulking surgery.

### 4.5. Lentiviral Particles

shMACC1 MISSION^®^ Lentiviral Transduction Particles (SHCLNV, Sigma Aldrich) are commercially available and used for the integration of MACC1-specific shRNA (pLKO.1 plasmid) into the genome of HGSOC PDOs to knock down MACC1. The concentration was 2.7 × 10^7^ VP/mL according to the datasheet, and viruses were thawed for the first time for this experiment without further quantification (for further information, see manufacturer instructions).

The plasmid vectors contain all genetic components for infection and stable insertion into the genome of the organoids, as well as an antibiotic resistance cassette (puromycin). The genetic target sequence of the construct is “CATTGAACTTTAGCAACTATG”. Transduced organoids were selected under puromycin selection pressure. Though in this case, we have used pre-made ready lentiviral particles, custom-made lentiviruses can be produced in the lab using transient transfection of envelope, packaging, and transfer plasmids to the 293T cells [12].

## 5. Procedure

### 5.1. Preparation of the Seeding Plate Beforehand (10 min)

- Note: The plate needs to be prepared beforehand so the BME Type 2 can solidify.

For each transduction condition, the bottom of one well of a 24-well non-tissue culture-treated plate needs to be covered with a 1:1 mixture of ADF++ (Advanced DMEM infused with 5% Glutamax (100×), 5% HEPES (1 M)) and BME Type 2, 80 µL per well total. The following procedure describes three transduction conditions (shMACC1, shControl, Mock).

1. Thaw 150 µL of BME Type 2 on ice (3 h) or overnight at 4 °C.
2. Place a 24-well plate on ice and make sure it is cold.
3. In the meantime, mix 125 µL of cold ADF++ with 125 µL BME Type 2 (1:1). Try to keep the mixture as cold as possible.
4. Pipet 80 µL of the mixture into the destined wells, fully coating the bottom of the wells. Take the plate off the ice and place it on an even surface.
5. When BME Type 2 is solidified, incubate the plate at 37 °C overnight.

### 5.2. Step 1: Preparation of the Organoids for the Lentiviral Transduction (1 h)

For optimal lentiviral transduction, depending on the growth and size of the organoids 3-4 full-grown wells in 24-well format need to be singularized for approximately 600.000 cells (~200.000 cells per condition are needed). Here, the protocol from Trillsch et al. [11] is followed. For the specific procedure of lentiviral transduction, the use of a 26-gauge syringe and a 200 µm filter for organoid singularization is recommended to minimize cell clumping.

- Note: All the following steps are conducted on the ice.

#### 5.2.1. Releasing the Organoids from Culture 

See “Passaging of Organoids, Biobanking, Long Term Cultivation” Steps 23–25 [11]. Briefly, organoids are retrieved from the BME Type 2 matrix by adding cold ADF++, followed by washing, and resuspending the pellet in 1 mL of TrypLE.

#### 5.2.2. Singularization of the Organoids

1. Incubate the cell suspension for 10 min in a water bath (37 °C) with intensive vortexing every 2–3 min.
2. **OPTIONAL STEP:** After the incubation, a 26-gauge syringe can be used to further disrupt big, aggregated cells. Some organoid lines may be sensitive to enormous stress triggered by syringing; therefore, try this step beforehand. In case only filtering is preferred, a higher overall number of cells is needed.
3. The suspension is then filtered through a 200 µm strainer into a new 15 mL falcon placed on the ice.
4. Add 2 mL of cold ADF++.
5. Centrifuge for 5 min at 300 g and discard the supernatant.

#### 5.2.3. Counting of the Cells

1. Add 3 mL of cold ADF++ to the cell pellet and mix.
2. Count the cells using an automatic cell counter or a Neubauer chamber. Use 200.000 cells per condition (three conditions).
• Note: Choosing an adequate number of cells, considering cell loss, around 1000 cells/µL BME Type 2 are seeded. We seed 200.000 cells into three wells (24-well-format, 50 µL BME Type 2, ~50.000 cells per well), ensuring a good density for growth, as density plays an important role in the formation and growth of the organoids. If you have fewer cells, adapt the protocol in a way that about 1000 cells are diluted in 1 µL of BME Type 2.
3. Per the condition (shMACC1-KD, shControl, Mock), take the amount containing 200,000 cells diluted in ADF++ and pipet into three falcons.
4. Centrifuge for 5 min at 300 g and discard the supernatant.
5. The pellet can be stored on ice (4 °C) for up to 3 h.

### 5.3. Step 2: Lentiviral Transduction (20 min)

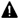
 **CRITICAL STEP** The following steps must be conducted under institutional biosafety regulations (Biosafety Level 2) and equipped with laminar flow cabinets. Appropriate personal protection has to be available for the person conducting the experiments.

#### 5.3.1. Preparation and Addition of the Virus (30 min)

- The virus is kept at −80 °C and put on ice for slow thawing.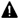 **CRITICAL STEP:** Do not vortex the virus, only centrifuge.
- Eppendorf tubes of 500 µL are prepared and labeled appropriately to aliquot the MACC1 and the control viral particles.
- Transduction medium (TM): Prepare 800 µL of organoid medium and add Polybrene in a concentration of 10 µg/mL. Add 200 µL of TM to each of the three conditions-pellets and mix generously by pipetting.
- A Multiplicity of Infection (MOI) of 1 is used for the shMACC1-KD as well as for shControl (Table 4). Add the volume needed and mix thoroughly by pipetting.
- Note: The MOI depends on the individual virus batch. Follow the suggestion made by the supplier.

**Table 4:** Overview of the three transduction conditions.

	shMACC1	shControl	Mock
Number of cells	200,000	200,000	200,000
Volume of TM [µL]	200	200	200
Type Viral particles	shMACC1	shControl	-

- Pipet the organoid mixtures into the prepared wells and label them on the plate. Incubate for 20 h at 37 °C.
- Note: The remaining virus should be aliquoted in 200 µL Eppendorf tubes since repetitive thawing may impact the potency of the virus.
- Note: The amount of BME Type 2 appropriate for the cell number used should be put on ice and left to thaw either overnight or for at least 3 h before continuing transduction the next day.

#### 5.3.2. Seeding of the Organoids (The Next Day; 2 h)

1. A 15 mL falcon is labeled and placed on ice for each of the three conditions.
2. Prewarm a labeled 24-well plate at 37 °C.
3. Keep the organoid media at room temperature.
4. Scrape up the organoid-virus mixture with the pipet and transfer all of it into the respective falcon.
5. Wash the well thoroughly with 1 mL cold ADF++ and transfer it into the respective falcons.
6. After centrifuging for 5 min at 300 g, the supernatant is discarded, and the pellets are washed with 1 mL cold ADF++ by pipetting thoroughly.
7. After centrifuging for 5 min at 300 g, the supernatant is removed, and the pellet is carefully diluted in 150 µL cold BME Type 2 (here, use the appropriate amount of BME Type 2 necessary for the cell number used).
• Note: Try to remove as much ADF++ as possible to avoid fragility of the BME Type 2 droplets and consecutive poor organoid growth. If necessary, repeat centrifugation at 350 g for 5 min.
8. Seed three wells of organoids diluted in 50 µL BME Type 2 per condition.

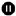

**PAUSE STEP:** Incubate the plate at 37 °C for 30 min.
9. Add 500 µL of organoid medium per well (for further information on organoid media composition, see Trillsch et al. [11]).

### 5.4. Step 3: Puromycin Selection of the Transduced Organoids and Longtime Cultivation

Puromycin selection is started on Day 2 after seeding the organoids (see Section 5.3.2.). Puromycin is diluted at 1 µg/mL in the respective organoid media, and 500 µL per well is added. Medium change with puromycin is repeated twice with the accompanying medium change.

### 5.5. Expansion of the Modified Organoids

The organoid cultures are passaged and expanded, as described in Trillsch et al., with a medium change every 3–4 days [11].

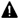
 **CRITICAL STEP:** The timing of organoid passaging depends on the individual growth and viability of the respective organoid line, as the conducted gene modification, infection efficiency, and passaging itself impact growth and viability to a varying degree.

### 5.6. Step 4: Isolation of RNA and Protein for Knockdown Validation via qPCR and Western Blot

#### 5.6.1. RNA Isolation

• Note: To ensure that the yield of RNA extraction is sufficient, at least 2-3 full-grown vital wells should be selected (Day 10-12 of growth). The control and the KD-Organoids should be synchronized, and RNA should be isolated on the same day.
1. Release the organoids from BME Type 2 as previously described (see Section 5.2.1)
2. Centrifuge the suspension for 5 min at 300 g and discard the supernatant.
3. Wash with 3 mL cold ADF++, centrifuge for 5 min at 300 g, and discard the supernatant.
• Note: Here, it is very important to work on ice to dissolve the BME Type 2 fully, as it will impact the quality of the RNA.
4. Wash with 4 mL cold PBS and centrifuge for 5 min at 600 g and remove the supernatant.
• Note: OPTIONAL STEP: If the pellet is cloudy, centrifuge again at 600 g.

#### 5.6.2. RNA Purification

• **Note:** As RNA is not stable at room temperature, be sure to conduct the full procedure on ice.
1. Prepare, in advance, a solution of β-Mercapto-Ethanol (RT) mixed with RLT-Buffer (1:100, RT); 350 µL/sample is needed.
2. The organoid pellet is diluted in 350 µL β-Mercapto-Ethanol under intense mixing.
3. With a 26-gauge syringe, the solution is mechanically disrupted and drawn up 2–3 times.
4. Add 350 µL of 70% ethanol and mix again.
5. The RNA is purified according to instructions by QIAGEN using the RNeasy Kit, 2021.
6. Load everything onto an RNeasy spin column and proceed.
7. The RNA is eluted using 30 µL of nuclease-free water, and the concentration of the RNA is measured using an Implen Nanophotometer.
• Note: The concentration of the purified RNA can vary between 10 and 400 ng/µL. Using 30 µL of nuclease-free water ensures that the concentration after elution is as high as possible.

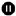

**PAUSE STEP:** The RNA is stored at −80 °C until further use.

#### 5.6.3. Reverse Transcription

For the two-step quantitative reverse transcription PCR (RT-qPCR), RNA samples are converted to complementary DNA (cDNA) using the cDNA Synthesis Kit (Biozym). Here, 1000 ng RNA is used as a template. This kit includes a cDNA synthesis buffer, RNase inhibitor, reverse transcriptase enzyme, nucleotide mix, and primers, all used according to the manufacturer’s instructions. Samples can be stored at −20 °C after dilution in nuclease-free water.

#### 5.6.4. Reverse Transcription Real-Time Quantitative PCR (RT-qPCR)

Using the TaqMan System for 3-phosphate dehydrogenase (GAPDH, endogenous control) and MACC1 (target gene), amplification is conducted in technical triplicates of each biological sample using a total reaction volume of 10 µL per reaction (Table 5). For the internal control, water is used. For quantification of gene expression, GAPDH is used.

The PCR was executed following the Applied Biosystems™ 7500 Fast Real-Time PCR System conditions (Table 6) with the detection of fluorescent signal, and the obtained data are analyzed by using the 7500 Fast SDS v1.5.1 Software (Applied Biosystems™).

Expression levels of the *KD* gene relative to an endogenous reference gene are quantified by calculation of the mean *Ct* values. Relative quantification (*RQ*) is calculated according to the following equations:

(1) $$\Delta Ct\left(KD\;sample\right)=\bar{Ct}\left(KD\;gene\right)-\bar{Ct}\left(reference\;gene\right)$$

(2) $$\Delta Ct\left(\mathrm{control}\right)=\bar{Ct}\left(tKD\;gene\right)-\bar{Ct}\left(reference\;gene\right)$$

(3) $$\Delta \Delta Ct=\bar{\Delta Ct}\left(KD\;sample\right)-\bar{\Delta Ct}\left(control\right)$$

(4) $$RQ=2^{-\Delta \Delta Ct}$$

#### 5.6.5. Protein Sample Preparation

1. Prepare in advance: mix 2 × Laemmli Buffer with β-Mercapto-Ethanol (1:50).
• The amount of Laemmli+β-Mercapto is highly dependent on the pellet size. For three full-grown wells, approximately 100–120 µL are needed.
2. Preheat a shaker to 96 °C.
• Isolation of protein is performed according to steps 1–4 of RNA isolation (see Section 5.6.1.), resulting in an organoid pellet.
3. Dilute the pellet in an appropriate amount of PBS. This is the same amount as the amount of Laemmli+β-Mercapto-Ethanol which will be used in Step 4.
4. Add 2 × Laemmli Buffer + β-Mercapto-Ethanol (1:1) and mix well.
5. **OPTIONAL STEP:** Use a 26-gauge syringe to further disrupt the organoids.
6. Transfer the mixture into a 1.5 mL low Protein-bind Eppendorf tube and place it in the preheated shaker for 10 min.

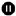

**PAUSE STEP:** Store the protein at −80 °C until further use.

#### 5.6.6. Western Blot Analysis

The lysates are prepared as previously described in Section 5.6.5. The KD samples and controls (14 µL per sample) are loaded onto a 10% polyacrylamide gel (thickness 1.5 mm) and separated by gel electrophoresis (70 V for 15 min, 100 V for 100 min). A semi-dry transfer to a 0.2 µm pore-sized PVDF membrane (Bio-Rad) was conducted using the Trans-Blot Turbo Transfer System. After blocking (1% bovine serum albumin (BSA), 3% milk powder) for 1.5 h at RT, incubation with primary antibodies, MACC1 (1:1250), β-Actin (1:500) is conducted at 4 °C overnight, where β-Actin acts as a control for normalization. Detection is performed by the incubation with the respective HRP-secondary antibodies (1:1000) for 1 h at RT followed by detection with Amersham ECL Prime Western Blotting Detection Reagent on the ChemiDoc MP Imaging System.

## 6. Results

**Figure 2:**
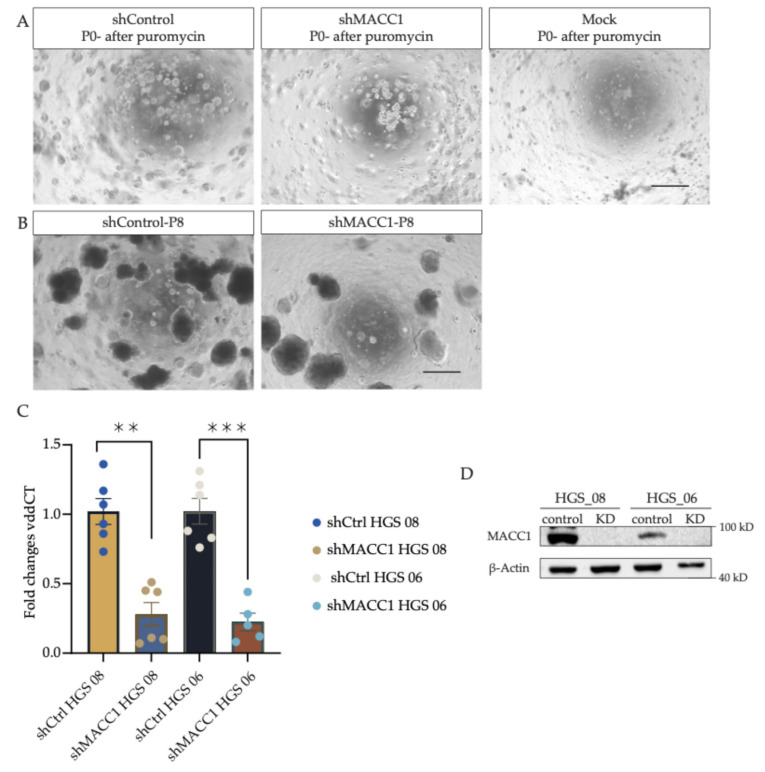
Depletion of MACC1 affects transcriptional and protein levels in transduced organoid lines. (**A**) HGS_06 after puromycin treatment. Comparison of the three conditions, shControl, shMACC1-KD, and Mock, after puromycin treatment (1 mg/mL) on Day 10 after lentiviral transduction. (**B**) HGS_06 shControl and shMACC1 KD after 2 months of organoid cultivation. The mock sample, as expected, underwent complete growth arrest under puromycin treatment and could not be expanded. Scale bar 200 µm. (**C**) qPCR of relative MACC1 expression levels in the long-term culture of transduced organoid lines. Individual data points represent independent biological replicates. Taken at 1-, 2- and 3-months post-transduction. Fold changes are calculated based on the vddCT values normalized to the control organoid line. Error bars are +-SEM. ** *p* = 0.0022 and *** *p* = 0.0006 in paired student *t*-test. (**D**) MACC1 knockdown as an effect of shMACC1 introduction was confirmed in both organoid lines by Western Blot analysis.

After lentiviral transduction, the successfully transduced organoids are selected with puromycin (7 days), as organoids in which integration in the genome did not occur show stress-related impaired growth and undergo cell death (see Figure 2A, mock control, upper right). In the first passage, after lentiviral transduction, the shMACC1 organoids showed slightly slower growth, potentially due to the puromycin selection, effects of gene silencing, and the process of transduction; however, after adequate passaging, they exhibited a robust long-term growth pattern, indicating that MACC1 is not indispensable for the organoid propagation (see Figure 2B Passage 8, post-transduction).

In both transduced organoid lines, a strong MACC1 KD could be achieved. A KD efficacy on an mRNA level for HGS 08 of 72% and 78% for HGS 06 was determined (relative to control transduction and normalized to GAPDH as internal control) (see Figure 2C). The samples were taken in pairs (shControl and shMACC1) and as independent biological replicates at 1, 2, and 3 months post-transduction, confirming the long-term stability of the downregulation. In agreement with this, the MACC1 band was not detectable at the protein level at 2 months post-transduction (loading control β-Actin; see Figure 2D; see Supplementary Materials Figure S1 for unprocessed blot data). The KD MACC1 organoids could be fully expanded and frozen as a stock and stored in liquid nitrogen for further experimentation.

## 7. Discussion

HGSOC-PDOs serve as a reliable model to investigate signaling pathways and tumor pathobiology in vitro [3]. In the search for potential novel biomarkers for early detection and disease management [17], we here show, for the first time, the successful lentiviral integration of shRNA targeting MACC1 into the genome of two high-grade serous ovarian cancer organoid lines. The integration of shControl, which is a non-targeting sequence, acts as a control for the unspecific collateral consequences that may be caused by viral transduction. As a negative control for the drug-based selection, mock organoids were treated with the same conditions as the transduced organoids to be able to interpret the effects of the transduction. Moreover, the long-term stability of the knockdown is validated by Western Blot at 2 months and RT-qPCR at 3 months post-transduction. Notably, the general phenotype of the transduced organoids remained unchanged.

Taken together, this protocol outlines a straightforward and easy-to-implement method to obtain HGSOC-PDO lines with depleted expression of the gene of interest. As a tool of choice, it remains an important option to complement other forms of genetic manipulations in organoid cultures, such as CRISPR editing via nucleofection. This approach has its own unique set of challenges, such as selection pressures and editing efficiency problems [18]. With each method of genetic manipulation having its individual effects on genomic regulation, shRNA knockdown in HGSOC-PDO is extremely helpful in strengthening the experimental design of studies in late preclinical research.

## Figures and Tables

**Table 5:** TagMan^®^ reaction components.

Reaction Components	Volume [µL]
TagMan^®^ Universal PCR Master Mix	5
TagMan^®^ Gene Expression Assay	0.5
cDNA sample (diluted 1:5)	2.5
RNase free Water	2

**Table 6:** RT-qPCR conditions.

Cycle	Temperature [°C]	Time [s]
1	90	20
40	95	3
	60	30

## Data Availability

All generated data are presented within this article. No new data sets were created during this study.

## Supplementary Materials

The following supporting information can be downloaded at https://www.mdpi.com/article/10.3390/mps7060104/s1: Figure S1: Western Blot unprocessed data.
