# Supplementary figures and images for: Establishment of Stable Knockdown of MACC1 Oncogene in Patient-Derived Ovarian Cancer Organoids
Source: Methods Protoc. 2024 Dec 20;7(6):104. doi: 10.3390/mps7060104 (PMC11678392; doi:10.3390/mps7060104)

Supplementary Data for Figure 2:

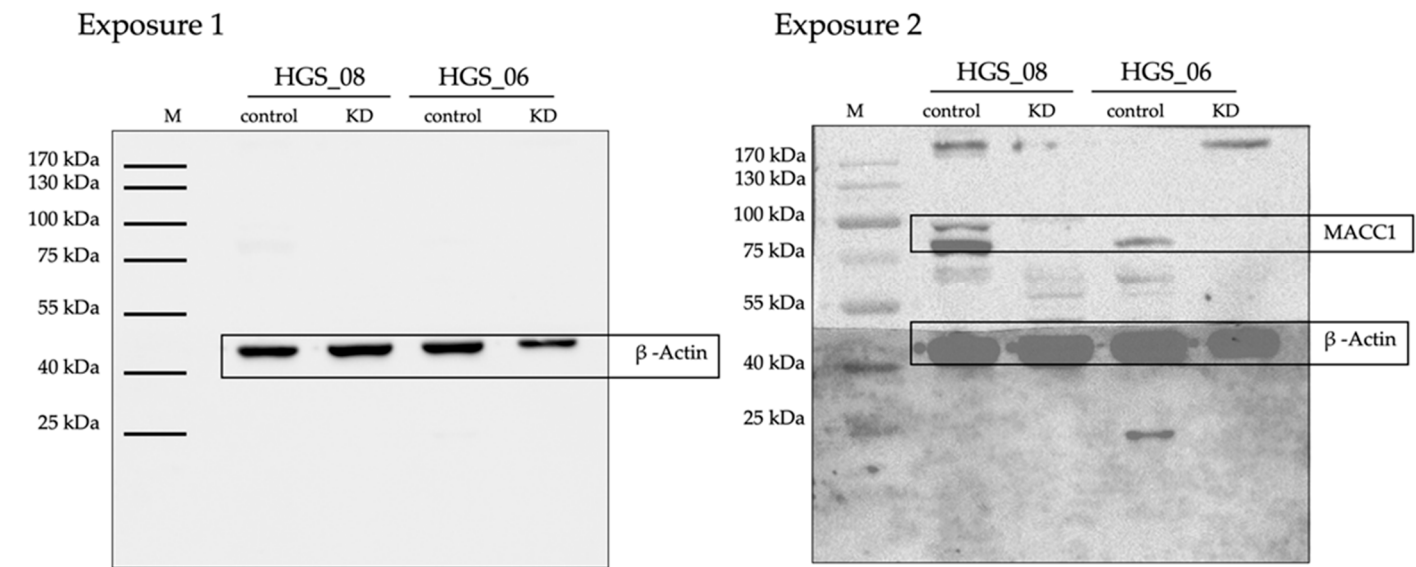

Figure S1. Western Blot unprocessed data.

Supplement: Supplementary file 1 [file mps-07-00104-s001.zip › mps-3269206-supplementary.pdf]
